# Longitudinal Assessment of OCT-Based Measures of Foveal Cone Structure in Achromatopsia

**DOI:** 10.1167/iovs.65.4.16

**Published:** 2024-04-08

**Authors:** Garrett Grissim, Ashleigh Walesa, Hannah M. Follett, Brian P. Higgins, Kaitlin Goetschel, Heather Heitkotter, Joseph Carroll

**Affiliations:** 1 School of Medicine, Medical College of Wisconsin, Milwaukee, Wisconsin, United States; 2 Ophthalmology and Visual Sciences, Medical College of Wisconsin, Milwaukee, Wisconsin, United States; 3 Cell Biology, Neurobiology, and Anatomy, Medical College of Wisconsin, Milwaukee, Wisconsin, United States

**Keywords:** achromatopsia (ACHM), cone photoreceptor, imaging, gene therapy, retina

## Abstract

**Purpose:**

Achromatopsia (ACHM) is an autosomal recessive retinal disease associated with reduced or absent cone function. There is debate regarding the extent to which cone structure shows progressive degeneration in patients with ACHM. Here, we used optical coherence tomography (OCT) images to evaluate outer nuclear layer (ONL) thickness and ellipsoid zone (EZ) integrity over time in individuals with ACHM.

**Methods:**

Sixty-three individuals with genetically confirmed ACHM with follow-up ranging from about 6 months to 10 years were imaged using either Bioptigen or Cirrus OCT. Foveal cone structure was evaluated by assessing EZ integrity and ONL thickness.

**Results:**

A total of 470 OCT images were graded, 243 OD and 227 OS. The baseline distribution of EZ grades was highly symmetrical between eyes (*P* = 0.99) and there was no significant interocular difference in baseline ONL thickness (*P* = 0.12). The EZ grade remained unchanged over the follow-up period for 60 of 63 individuals. Foveal ONL thickness showed a clinically significant change in only 1 of the 61 individuals analyzed, although detailed adaptive optics imaging revealed no changes in cone density in this individual.

**Conclusions:**

ACHM appears to be a generally stable condition, at least over the follow-up period assessed here. As cones are the cellular targets for emerging gene therapies, stable EZ and ONL thickness demonstrate therapeutic potential for ACHM, although other aspects of the visual system need to be considered when determining the best timing for therapeutic intervention.

Achromatopsia (ACHM) is an autosomal recessive retinal disease affecting about 1 in 30,000 individuals.^1^ ACHM is caused by genetic mutations linked to six known genes (*CNGA3*, *CNGB3*, *GNAT2*, *PDE6C*, *PDE6H*, and *ATF6*); all of which, with the exception of *ATF6*, encode various components of the cone phototransduction cascade.^2^^–^^9^ Specifically, *ATF6* encodes for a transcription factor that has a key role in regulating cell homeostasis, and mutations can contribute to foveal hypoplasia,^2^ whereas *CNGA3* and *CNGB3* encode for the α and β subunits, respectively, of the cGMP gated cation channel and account for 80% of ACHM cases.^1^^,^^3^ Across genotypes, ACHM generally results in sequelae of reduced visual acuity, nystagmus, photophobia, and reduced or absent color discrimination.^1^

Structurally, patients with ACHM have an unremarkable fundus appearance, although rarely can exhibit retinal pigment epithelial (RPE) disturbance and/or atrophy.^10^^,^^11^ In contrast, the retina shows varying degrees of abnormal structure when examined with optical coherence tomography (OCT). Studies have documented variable degrees of foveal hypoplasia,^12^ variable disruption of the outer retinal layers,^12^ and diminished intensity of the second hyper-reflective outer retinal band.^10^^,^^11^^,^^13^ Although this band is interchangeably referred to as the ellipsoid zone (EZ), inner segment/outer segment junction (IS/OS) and inner segment ellipsoid (ISe), it is generally agreed to at least in part originate from the photoreceptor inner segments (we will herein use EZ). Gene therapy efforts have recently been translated from animal models of ACHM to phase I/II studies in humans for the *CNGA3* and *CNGB3* genes.^14^ Early trials have demonstrated tolerable and safe profiles for humans (Iannaccone A, et al. IOVS 2022;63:ARVO E-Abstract 2829).^15^^,^^16^ In addition, it has been reported that some patients show modest functional changes implicating improvement in the structure or function of cone photoreceptor pathways.^17^^,^^18^ Knowing the degree to which retinal structure in ACHM is either stable or degenerative is important as this can aid in interpreting results from longitudinal therapeutic trials as well as helping determine the optimum age for intervention.

There have been multiple cross-sectional and longitudinal studies seeking to establish the stable or progressive nature of ACHM (see the Table).^11^^,^^12^^,^^19^^–^^27^ Using similar spectral-domain OCT (SD-OCT) imaging methods, these studies have examined numerous measures of photoreceptor structure, including the relative disruption of the EZ band, width of the hyporeflective zone (HRZ), central macular thickness, foveal total retinal thickness (FTRT), and outer nuclear layer (ONL) thickness. Despite relatively large sample sizes in many of these studies and follow-up periods of up 5 years in some studies, there remains a lack of consensus regarding the stability of photoreceptor structure in patients with ACHM. Here, we sought to test the hypothesis that ONL thickness and EZ integrity remain stable over time in congenital ACHM.

**Table.** Comparison of Previous Study Demographics in Achromatopsia

Study	Type	N (% Children)^*^	Mean Follow-Up (Years)	Genotype	Conclusion
Thiadens et al. (2010)^19^	Cross-sectional	40 (30%)	N/A	*CNGA3* (2)*CNGB3* (33)*PDE6C* (5)	Loss of inner and outer cone segments were present in 28 (70%) of patients, the majority of cone loss appeared during the second decade of life. Retinal thickness decreased with increasing age (*P* = 0.011).
Thomas et al. (2011)^11^	Cross-sectional	13 (53.8%)	N/A	N/A	The presence of an HRZ and significance of ONL thinning was correlated to age, *P* = 0.001 and *P* = 0.002, respectively.
Thomas et al. (2012)^20^	Longitudinal	8 (62.5%)	1.3	*CNGA3* (5)*CNGB3* (2)Unknown (1)	Five (62.5%) patients had worsening morphologic changes at the EZ and a decrease in ONL thickness with follow up. However, these five patients were <10 y.
Sundaram et al. (2014)^12^	Cross-sectional	40 (40%)	N/A	*CNGA3* (18)*CNGB3* (15)*GNAT2* (4)*PDE6C* (1)Unknown (2)	No correlation between age and structure or function was observed.
Aboshiha et al. (2014)^21^	Longitudinal	38 (39.5%)	1.6	*CNGA3* (18)*CNGB3* (13)*GNAT2* (4)*PDE6C* (1)Unknown (2)	Two (5%) patients progressed to a worse OCT grade during follow-up, though FTRT, ONL thickness, HRZ diameter, visual acuity, contrast sensitivity, mean retinal sensitivity, and fixation stability showed no significant change during follow-up.
Zobor et al. (2017)^22^	Cross-sectional	36 (19.4%)	N/A	*CNGA3* (36)	No correlation between age and visual function or retinal structure was observed.
Langlo et al. (2017)^23^	Longitudinal	41 (56.1%)	1.07	*CNGB3* (41)	Seven (17%) patients progressed to worse OCT grade during follow-up. A small but statistically significant *increase* in ONL thickness was observed. However, peak cone density remained unchanged with follow-up.
Hirji et al. (2018)^24^	Longitudinal	50 (42.0%)	5.1	*ATF6* (4)*CNGA3* (20)*CNGB3* (23)*GNAT2* (2)*PDE6C* (1)	Six (12%) patients progressed to a worse OCT grade, however, FTRT and HRZ diameter remained stable during follow-up. A small but statistically significant *increase* in ONL thickness was observed.
Brunetti-Pierri et al. (2021)^25^	Longitudinal	21 (71.4%)	5.4	*CNGA3* (7)*CNGB3* (5)*GNAT2* (3)*PDE6C* (1)Unknown (5)	Two (12.5%) patients progressed to a worse OCT grade during follow-up, although central retinal thickness did not change significantly during follow-up.
Tekavčič Pompe et al. (2022)^26^	Longitudinal	11 (81.8%)	7.7 (4.7 for OCT)	*CNGA3* (6)*CNGB3* (5)	Only three patients progressed on OCT grade.
Triantafylla et al. (2022)^27^	Longitudinal	17 (29.4%)	5.7	*CNGA3* (11)*CNGB3* (6)	Fifteen (88%) patients progressed to a worse OCT grade during follow-up, though ONL thickness showed no significant change during follow-up.
This study	Longitudinal	63 (54%)	3.1	*ATF6* (1)*CNGA3* (8)*CNGB3* (54)	Three (5%) individuals progressed to worse OCT grade and only one (2%) had a significant decrease in ONL thickness.

* Children defined as individuals under 20 years of age.

ACHM – achromatopsia, EZ – ellipsoid zone, FA – fundus autofluorescence, FTRT – foveal total retinal thickness, HRZ – hyporeflective zone, OCT – optical coherence tomography, ONL – outer nuclear layer.

## Methods

Sixty-three individuals with genetically confirmed (8 *CNGA3*, 54 *CNGB3*, and 1 *ATF6*) ACHM were included in this study. Refer to Supplementary Table S1 for information regarding prior reporting of results from these individuals. This research followed the tenets of the Declaration of Helsinki and was approved by the Institutional Review Board (IRB) at the Medical College of Wisconsin (PRO00030741). Written informed consent was obtained from all individuals prior to data collection and their images and data stored in an IRB-approved data bank.

### Optical Coherence Tomography 

Images used in this study were acquired using either the Bioptigen Spectral Domain OCT (Leica Microsystems, Deerfield, IL, USA) or Cirrus high-definition OCT (HD-OCT; Carl Zeiss Meditec, Dublin, CA, USA). For the Bioptigen OCT, the images consisted of horizontal and vertical line scans bisecting the fovea (nominal scan lengths were between 6–12 mm, with 1000 A-scans per B-scan and 80–120 B scans) and volume scans (nominal scan dimension of 3 × 3 mm, with 400 A-scans per B-scan and 400 B-scans per volume or nominal scan dimension of 7 × 7 mm, with 750 A-scans per B-scan and 250 B-scans per volume). If there was not a Bioptigen line or volume scan for an individual, then Cirrus HD line scans (nominal scan length of 6 mm) and Macular Cubes (512 A-scans, 128 B-scans; nominal scan size of 6 × 6 mm) were included. For our EZ analysis 470 images were utilized, OD: 243 and OS: 227. Of those 470, 410 were Bioptigen (line scans = 386 and volume scans = 24) and 60 were Cirrus (macular cube = 49 and HD line scans = 11). For our ONL analysis 445 of the 470 images were utilized, OD = 228 and OS = 217. Not all images were used as those images belonged to individuals with an OCT with extensive retinal and RPE atrophy and thus an ONL thickness of 0 mm. Of the 445 images used for ONL analysis, 386 were Bioptigen (line scans = 362 and volume scans = 24) and 59 were Cirrus (macular cubes = 48 and HD line scans = 11). In all cases, we endeavored to use the highest quality scan that was available to make the assessment (ONL thickness or EZ grade).

### OCT Processing

The raw Bioptigen line scans were processed using ImageJ.^28^ Each OCT line scan was manually inspected to identify the highest quality scan that transected the location of the incipient fovea. Once this B-scan was identified, it was then used as the reference frame which we aligned the remaining B-scans to via the TurboReg plugin.^29^ This was repeated for all OCT line scans. After the alignment, B-scans that were of poor quality or did not align well were discarded. With the remaining B-scans the Z-project function was used to get an average B-scan image (average number of B-scans used = 23.4 with a range of 2-120) which provided higher signal-to-noise ratio. For instances where a Bioptigen volume scan was used, the foveal B-scan was extracted along with immediately adjacent B-scans, which were then aligned and averaged using the same process as for the line scans. For the Cirrus scans, HD line scan images used were processed and averaged automatically by the onboard software, whereas for the macular cube volumes a single B-scan was manually chosen based on being the foveal-most scan in the volume (determined qualitatively by inspecting the extrusion of inner retinal layers, ONL thickening, and foveal reflex).

### OCT Analysis

**Figure 1.**
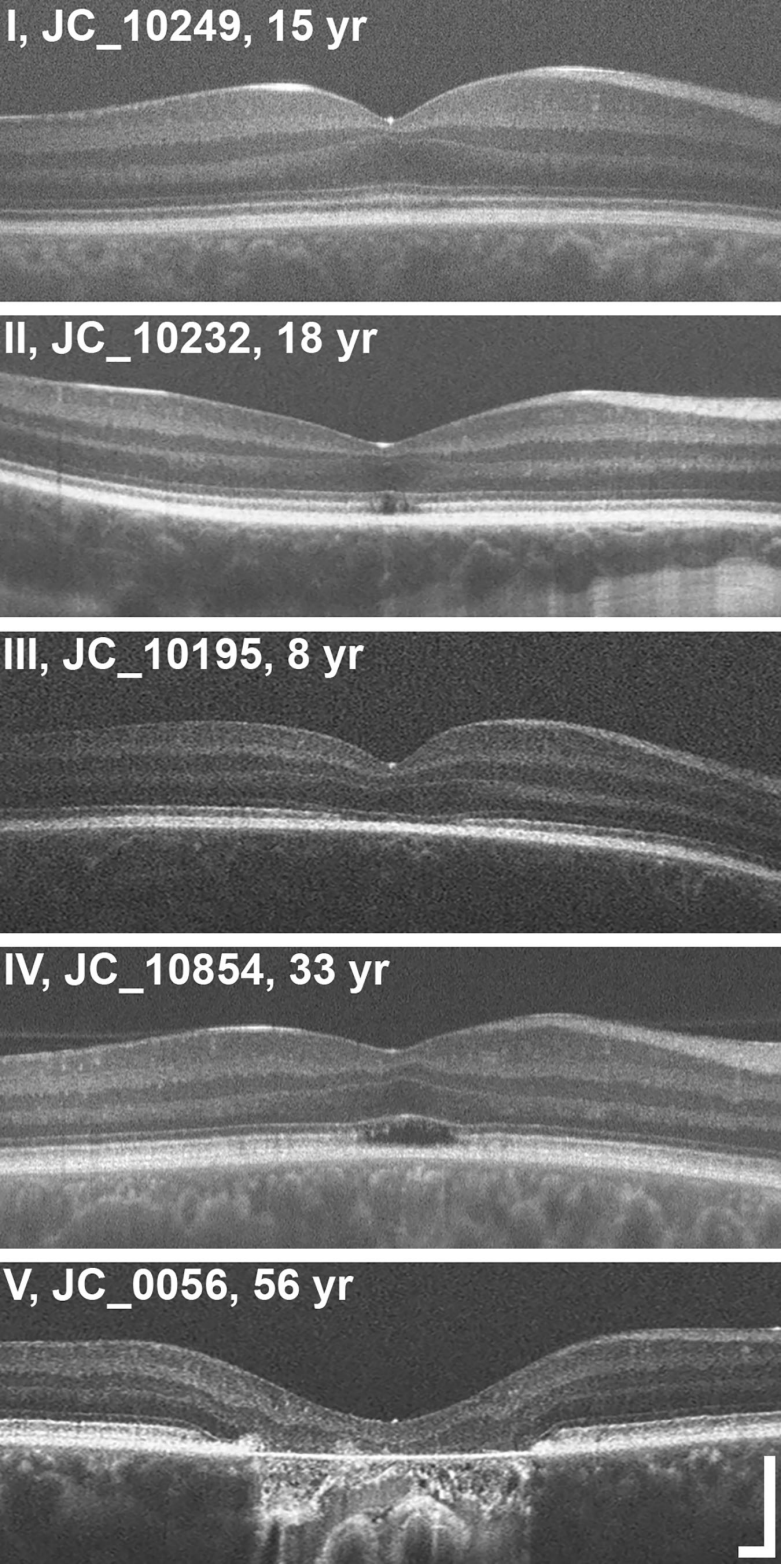
Illustrating the five different EZ grades on OCT. Scans were assigned one of five different grades: I - intact and continuous inner segment ellipsoid zone (EZ), II - disruption in the EZ, III - absence of the EZ, IV - hyporeflective zone (HRZ) present, or V - outer retinal atrophy and RPE. All five individuals in this figure had *CNGB3*-associated achromatopsia and the age listed is at the time of OCT scan acquisition. Scale bars = 200 µm.

Using a previously established grading scale, the EZ band at the foveal region of each OCT image was first graded on a scale of I to V.^12^ Grade I is an intact and continuous EZ, grade II is a disruption in the EZ, grade III is an absence of the EZ, grade IV represents that HRZ is present, and grade V is the outer retinal atrophy and RPE loss. These grades may not represent the sequential disease sequence of ACHM,^26^ but do serve as a reliable way to qualitatively categorize individual scans. See Figure 1 for visual examples of the different OCT grades. Initial grading was performed twice in a masked fashion by a single observer (author G.G.). For images with conflicting foveal grades (*n* = 10), they were presented to a second grader in a masked fashion to determine final grade (author J.C.).

Outer nuclear layer (ONL) thickness was measured by a single observer (author G.G.) using the measurement tool in ImageJ to extract an average of three measurements. ONL thickness was defined as the distance (in µm) between the external limiting membrane (ELM) and either the internal limiting membrane (ILM), in eyes with normal foveal morphology, or the posterior boundary of the outer plexiform layer (OPL) in eyes with foveal hypoplasia. Paired *t* tests were used to examine OD and OS ONL thickness values (GraphPad Prism version 8; GraphPad Software, La Jolla, CA, USA). The ONL thickness difference (OD-OS) at baseline was further compared using a Bland-Altman plot.^30^ Finally, a linear regression examining the rate of change for ONL thickness (as a function of time) was calculated in Microsoft Excel (Microsoft, Redmond, WA, USA). Linear regressions were calculated for each individual and then normalized to the average ONL thickness at baseline.

## Results

### Demographics and Genetics

Our study consisted of 63 individuals, 34 males (54%) and 29 females (46%), who were all genetically confirmed to have ACHM (*CNGA3* = 8, *CNGB3* = 54, and *ATF6* = 1). Specific genotype information for all individuals is provided in Supplementary Table S2. The average age at the baseline visit was 23.03 years (range = 6.8–64.1 years). The number of imaging visits ranged from 2 to 9, with follow-up ranging from 0.44 years and 10.02 years (average age was 26.29 years at the last visit). Of our 63 individuals, 34 (54%) were in their first 2 decades of life (0–20 years). Of those 34 individuals, 11 were 0 to 10 years and 23 were 10 to 20 years (at the initial visit). For individuals in their first 2 decades of life, the mean (± SD) age was 12.66 ± 3.67 years, with a mean (± SD) follow-up of 2.75 ± 2.60 years for OD and 2.67 ± 2.49 years for OS.

### Baseline Assessment

**Figure 2.**
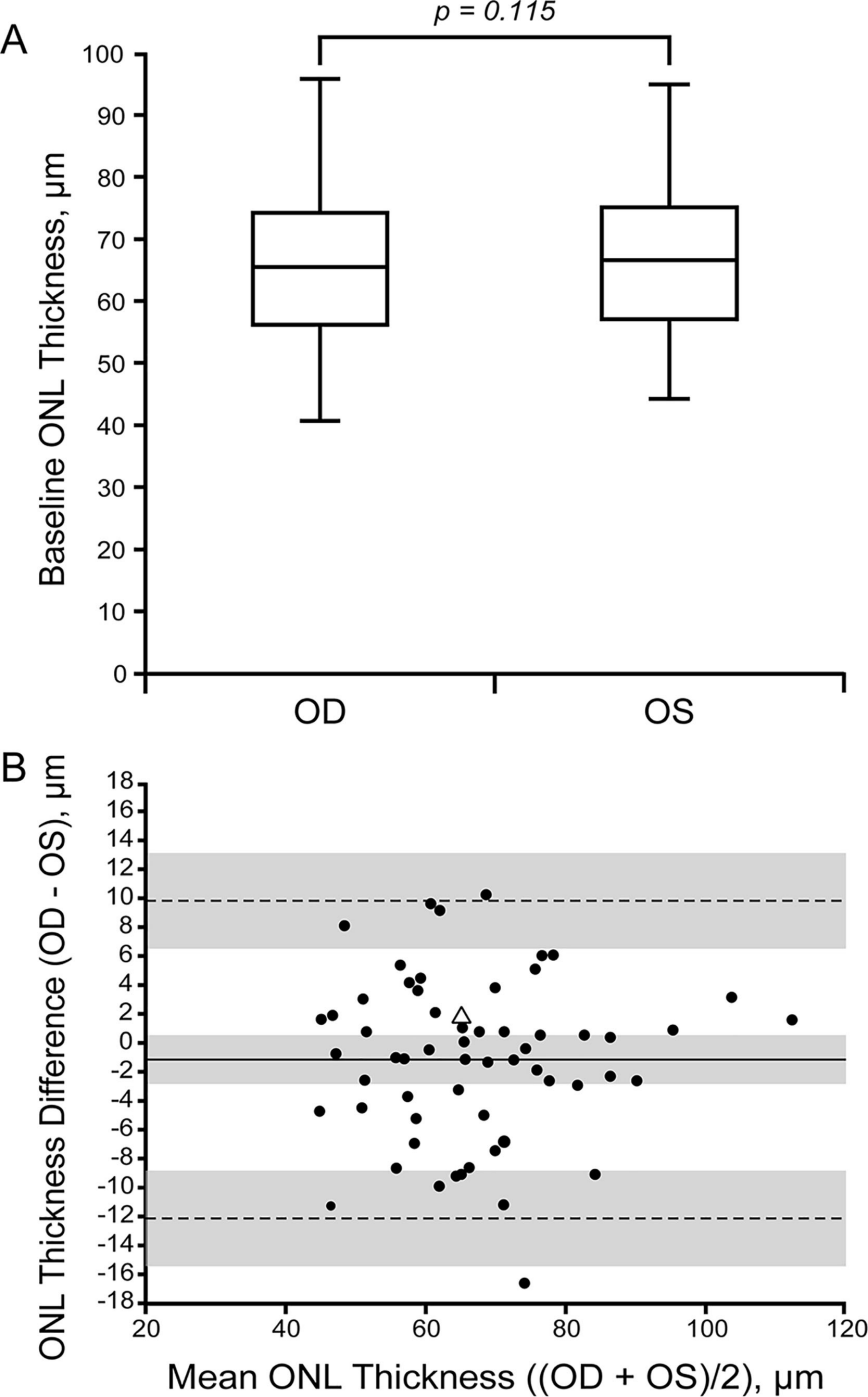
Foveal ONL thickness at baseline in ACHM. Shown is a box and whisker plot (**A**) and a Bland-Altman plot (**B**) for baseline ONL thickness between OD and OS. For the box and whisker plot the minimum and maximum values are represented, along with the 25th, 50th, and 75th percentiles. For the Bland-Altman plot, the *dashed lines* represent the 95% limits of agreement, the *solid line* represents the average bias between eyes, and the *gray shaded* regions represent the 95% confidence intervals for the bias and limits of agreement. The triangle represents the individual that showed a significant decrease in ONL thickness over time (see Fig. 5). In a previous study, mean ± SD ONL thickness in 42 individuals with normal vision was 112.9 ± 15.2 µm (OD) and 112.1 ± 13.9 µm (OS).^32^

At baseline, the distribution of EZ grades across all 63 individuals was symmetrical between eyes (*P* = 0.99, Chi-square test): OD: I = 8 (13%), II = 33 (52%), III = 2 (3%), IV = 17 (27%), V = 3 (5%); OS: I = 8 (13%), II = 32 (52%), III = 2 (3%), IV = 17 (28%), V = 2 (3%). Our baseline distribution of EZ grades was similar to that reported in previous studies.^21^^,^^24^^,^^25^^,^^27^^,^^31^ For ONL analysis, not all eyes from our studied cohort were included, with 60 (OD) and 61 (OS) eyes included in the analyses. This is because eyes with an OCT grade of V (*n* = 3) have an ONL thickness of 0 µm, which may introduce a floor effect and skew the assessment of symmetry of ONL thickness. The reason for the difference in the number of OD and OS eyes is because one individual had differing OCT grades between OD and OS (OD = V and OS = II). Of the original 470 OCT images, 445 (95%) images were analyzed for ONL thickness. All 25 images not graded belonged to those individuals with an OCT grade V. Mean ONL thickness at the first imaging date was 66.40 µm (range = 27.06 to 113.19 µm) and 67.37 µm (range = 34.58 to 111.62 µm) for OD and OS, respectively (Fig. 2A). These values are comparable to previous studies in other ACHM cohorts,^11^^,^^23^^,^^24^^,^^27^ and below previously reported normative values.^32^ The baseline mean ONL thickness values for OD and OS were not statistically different in our cohort (*P* = 0.115, paired *t*-test). The interocular symmetry for individual eyes can be visualized in the Bland-Altman plot in Figure 2B. Differences (OD-OS) ranged from 10.20 µm to −16.66 µm. The mean bias (OD-OS/2) was −1.16 µm, with a 95% confidence interval (CI) of 0.29 µm to −2.61 µm. The upper and lower limits of agreement were 9.81 µm and −12.13 µm, respectively.

### Longitudinal Assessment

**Figure 3.**
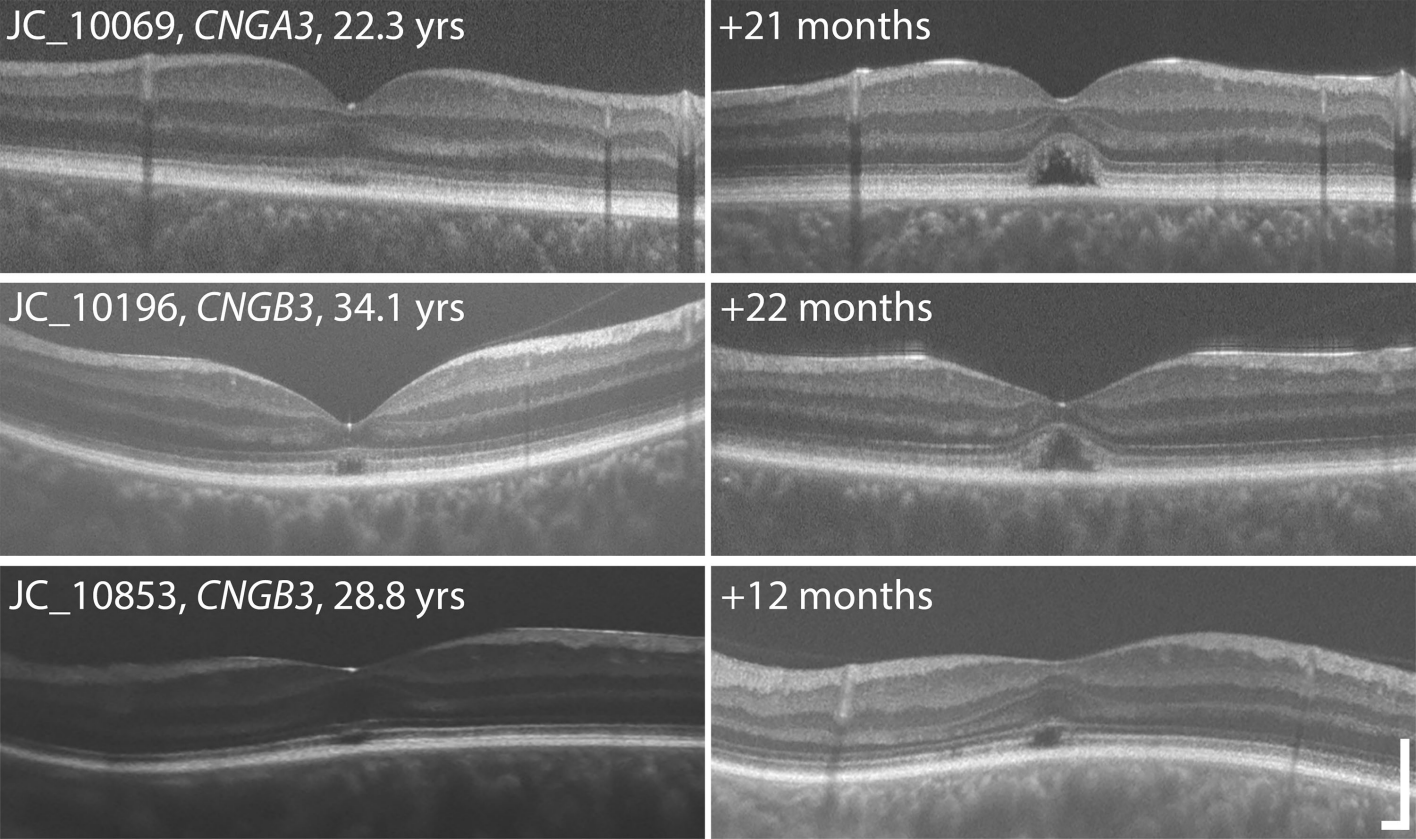
OCT images from three of the five eyes that showed a change in EZ grade (OD: *n* = 3 and OS: *n* = 2). These eyes belonged to three individuals and the images shown are vertical scans from their right eyes. The three individuals shown all represent a change of OCT grade from a two to a four. A grade II is represented by a disturbance or disrupted EZ and a grade IV is the denoted by the presence of the HRZ. Age on the scans in the *left column* represent the age at the time of OCT scan acquisition, with the follow-up time provided on the *right column* of scans. Scale bars = 200 µm.

For our EZ analysis, the mean follow-up period was 3.26 and 2.95 years for OD and OS, respectively. Over this period, a total of five eyes showed a change in grade (OD: *n* = 3 and OS: *n* = 2). These five eyes belonged to three individuals (two of the individuals had bilateral changes in OCT grade, whereas the third individual had a unilateral change; see Fig. 3). The changes observed in these individuals went from a grade II (EZ disruption) to a grade IV (HRZ) over 72, 34, and 13 months. Note that these time frames are not the total follow-up period for these individuals, rather it represents the difference from baseline to the first visit at which the OCT grade had changed. In all five eyes, the OCT grade remained stable after that point. For the remaining 60 individuals (95.2%), their initial OCT grade remained unchanged throughout the entire follow-up period.

**Figure 4.**
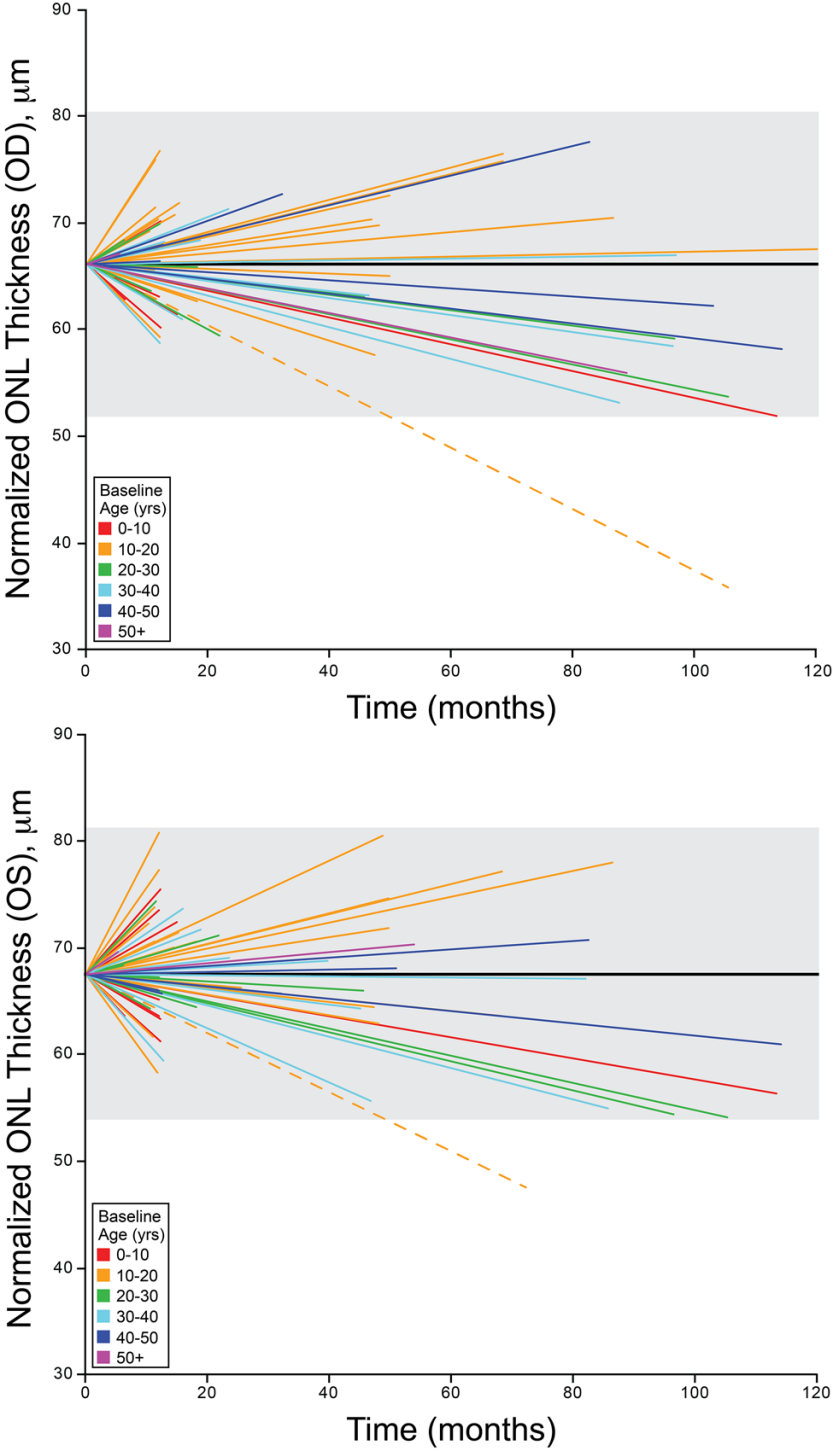
Foveal ONL thickness over time in ACHM. Shown are linear regression fits to the ONL thickness data for each individual for both the right eye (*top*) and left eye (*bottom*). The *solid black line* represents the average ONL thickness at baseline, 66.14 µm and 67.56 µm for OD and OS, respectively. The *shaded* region denotes expected repeatability (14 µm), based on previously published data.^32^ The *dashed line* represents the individual with the largest change in ONL thickness. Regression lines are color coded, as indicated based on the age at baseline imaging.

For our ONL analysis, the mean follow-up period was 3.17 and 2.82 years for OD and OS, respectively. Using our initial and final timepoints there was no significant difference in ONL thickness (paired *t*-test: t(59) = 0.99, *P* = 0.33 and t(60) = 0.09, *P* = 0.93 for OD and OS, respectively). The mean (95% CI) difference in ONL thickness between the first and last timepoints was −0.91 µm, 95% CI = −2.77 to 0.94 µm for OD and −0.07 µm, 95% CI = −1.81 to 1.66 µm for OS. To further evaluate our rate of change over time, the ONL thickness was plotted as a linear regression Figure 4. We used previously established repeatability values for this ONL thickness method (14 µm)^32^ to determine which individuals showed a clinically meaningful change in ONL thickness. Our linear regression model revealed one individual to have a significant change in ONL thickness over time (in both OD and OS), while the remaining OD = 59 (98%) and OS = 60 (98%) showed no significant change over time. There was no significant relationship between the regression slopes and age at baseline for either OD (*r* = 0.0066, *P* = 0.96) or OS (*r* = −0.029, *P* = 0.83).

**Figure 5.**
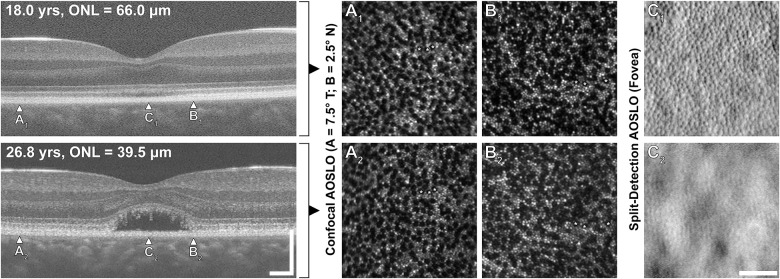
Assessing cone structure in an individual showing a change in OCT grade and ONL thickness. This individual (JC_10069) had an OCT grade change from grade II to grade IV, with a concomitant decrease in ONL thickness from 66.0 µm to 39.5 µm over the nearly 9-year follow-up period. Corresponding confocal AOSLO images (**A**, **B**) reveal stable parafoveal cone structure. Although the HRZ results in lower image quality of the foveal split-detection AOSLO image (**C_2_**), a contiguous mosaic of cone inner segments remains visible (despite the pronounced change in ONL appearance). The measured cone density values are significantly below values reported in normal retina (see Supplementary Fig. S1),^59^ but did not show significant change between the imaging sessions (see text). Scale bar on AOSLO images = 50 µm (applies to all 6 panels). Scale bars on OCT images = 200 µm.

The individual that showed a significant change in ONL thickness was one of the individuals observed to have a change in OCT grade (see Fig. 3; ID: JC_10069). To see if this significant change in ONL thickness was due to a degradation in cone structure, images of the photoreceptor mosaic obtained with adaptive optics scanning light ophthalmoscopy (AOSLO) were examined (see Supplementary Methods). These images were acquired from multiple prior studies,^33^^–^^35^ and not prospectively for this OCT study. We examined parafoveal (7.5 degrees and 2.5 degrees eccentricity) photoreceptor structure using the confocal AOSLO images, whereas foveal images were compared using the split-detection AOSLO images (due to the non-waveguiding nature of ACHM cones).^31^ We did not observe any loss in cone structure that accompanied the emergence of the HRZ, with cone structure remaining stable over the 9-year follow-up (Fig. 5). Foveal cone density was 18,541 cones/mm^2^ at baseline and 16,831 cones/mm^2^ at the follow-up visit (9.2% decrease), cone density at 2.5 degrees was 9515 cones/mm^2^ at baseline and 9470 cones/mm^2^ at the follow-up visit (0.47% decrease), and cone density at 7.5 degrees was 8955 cones/mm^2^ at baseline and 8707 cones/mm^2^ at the follow-up visit (2.76% decrease). These changes are comparable to previous repeatability measures in normal retina,^36^^,^^37^ and are thus not indicative of any significant change in cone structure.

## Discussion

Our OCT assessment showed that three (4.8%) individuals had a change in EZ integrity (assessed with OCT) across the follow-up period studied here. The mean follow-up for these individuals who demonstrated change was 4.83 years and the mean age at baseline and end follow-up was 26.6 and 31.5 years, respectively. However, the majority (95.2%) of our individuals showed stability of their EZ grades over time. These results are consistent with a stable course for ACHM. Except for one individual, foveal ONL thickness also showed no significant change over the follow-up period studied here. Interestingly, the one individual found to have a significant change in ONL thickness showed no change in cone structure as assessed with AOSLO imaging. This suggests that the apparent decrease in ONL thickness is not due to loss of cone structure, but rather compression of the ONL or displacement of cone nuclei by the emerging HRZ. This is important, as it indicates that ONL thickness may not be a reliable surrogate for cone density in ACHM and suggests using caution to describe ONL thinning as evidence of “cone degeneration.” Our data raise an important question as to why there remains discrepancy in the literature regarding progression in ACHM. We discuss below four main factors that we think could explain this.

### Genotypic Differences

ACHM has multiple genetic causes. Thus, an obvious possible explanation for differences between studies could be the genetic composition of their respective cohorts. One notable difference between our study and most of the studies outlined in the Table is that a large majority (86%) of our cohort had *CNGB3*-associated ACHM (see Supplementary Table S2). Additionally, some of the other studies included individuals with *ATF6*-, *GNAT2*-, and/or *PDE6C*-associated ACHM. There are reported differences in residual visual function across some ACHM genotypes,^38^^–^^40^ so it is important to consider underlying genotype when comparing studies and drawing conclusions that treat ACHM as a singular condition.

### Disambiguating Developmental Changes in Retinal Structure From Progression

Many previous studies (including ours) include a significant percent of children in their study population (ranging from 19.4–81.8%). With the advent of handheld OCT, examination of foveal structure is possible in neonates and young infants, even in ACHM.^41^ There are rapid foveal changes occurring during the postnatal period that can dramatically affect the appearance of the foveal region on OCT images.^42^^–^^46^ Relevant to grading of EZ disruption in ACHM is the observation that the outer hyper-reflective bands emerge rather late in retinal development.^43^^,^^47^ Diagnostic confounds are not limited to newborns, as although the retina may appear structurally similar to an adult by 17 months, it may not be fully developed until the second decade of life.^42^ For example, foveal cone density can increase as much as 10-fold over the first 10 years of development.^42^ Presumably, these same developmental mechanisms are present in ACHM, albeit acting on a smaller number of cone cells than in normal retinas. Thus, it is challenging to definitively assign retinal changes in ACHM retinas as being caused by disease progression (and not simply a reflection of normal anatomic development).

This confound can be demonstrated by reviewing images in a recent study from Triantafylla et al., where 15 (88%) of their 17 patients had an OCT grade change (interpreted as a worsening of retinal structure).^27^ However 10 of those 15 patients had baseline imaging before the age of 10 (average age = 5.7 years). In addition, their youngest subject (ID 12, 3 months of age at baseline), was documented to have a worsening grade change. However, inspection of the OCT scans appears to show an improvement in EZ integrity over time – the baseline image has an absent EZ, whereas 42 months later, the EZ is present with a focal disruption. Although there is technically a change in grade, we do not believe it is due to a degradation in cone structure. The emergence of the EZ would represent an improvement in cone photoreceptor structure, and this can be explained via previous studies concluding the presence of cone elongation and drastic increases in cone density during the ages of 0 to 10.^42^^–^^44^ It is also noteworthy to mention that only 11 (17%) patients from our cohort were in their first decade of life at their initial visit. It may be worth a comprehensive re-analysis of data from all these prior studies that excludes individuals over 20 years of age to eliminate the confound of retinal developmental changes.

### Disambiguating Age-Related Changes in Retinal Structure From Progression

Just as retinal development can confound interpretation of longitudinal and even cross-sectional OCT data in ACHM, so can normal age-related changes. As there are age-related change in all ocular structures,^48^ it is important to consider how these changes may impact the appearance of the ACHM retina (which is abnormal to begin with). There is no consensus for the photoreceptor layers, with reports of thinning of the parafoveal photoreceptor layers with age,^49^ thickening of the ONL with increasing age,^50^ and even no significant change with age.^51^ Histological data have identified changes in both rods and cones with age,^52^^–^^54^ so further in vivo imaging studies may be warranted to clarify what “normal” age-related changes exist (if any). This is important for future analyses of cone structure in ACHM, which should control for expected/normal age-related changes.

One example of where this confound could be affecting interpretation is the cross-sectional study of 40 patients by Thiadens et al.,^19^ who concluded that there is progressive cone loss in ACHM. This manifested as altered appearance of the photoreceptor layers (EZ or IS/OS) on SD-OCT and was suggested to begin primarily in the second decade of life. They also observed higher rates of RPE atrophy in their older patients and used this as evidence of progression. However, any group of individuals over 50 years of age would be expected to have a higher rate of RPE atrophy compared to a group of teenagers (due simply to the presence of age-related macular degeneration [AMD] in some of the older individuals). There is no reason to think that AMD would be less prevalent in patients with ACHM, so interpretation of longitudinal age-related changes cannot be conclusively attributed to the primary ACHM disease process.

### Acquisition Errors

A final contributing factor has to do with the challenges in imaging individuals with nystagmus – OCT images from individuals with ACHM can often have a number of artifacts.^34^ It is important to understand how these artifacts might alter our interpretation of what is really happening to specific retinal layers. This is especially relevant to measures of the HRZ width, as horizontal eye motion during B-scan acquisition can result in an apparent narrowing or widening of the HRZ. One might not expect this to cause systematic misinterpretation, but in cross-sectional studies of only one scan per individual or follow-up studies with only two timepoints, any such artifacts would obscure the truth. Additionally, as fixational stability has been reported to improve with age in ACHM,^55^ the frequency of these artifacts may be higher in younger cohorts.

For longitudinal studies, ensuring that scans capture the same retinal location can be challenging in individuals with ACHM. This is due to their above-mentioned nystagmus but also the non-foveal fixation locus that some individuals adopt. There is an example of scan misalignments between baseline and follow-up images in Triantafylla et al.,^27^ as their subject ID 11 has a prominent foveal reflex in the baseline image that is absent 6 years later in the follow-up image. The nerve fiber layer thickness is also different, suggesting the B-scans do not traverse the exact same retinal location. We endeavored to minimize these misalignments by utilizing the Bioptigen OCT where possible. Whereas the off-line processing required for these scans is not ideal for efficient workflow, it affords the opportunity to (1) select the “foveal-most” B-scan, and (2) select the B-scan that best aligns with images from other timepoints. It may be that moving away from a B-scan based analysis and more toward a volumetric approach could help avoid any errors due to scan misalignment, which may be more feasible as OCT devices increase their speed of acquisition. Alternatively, computer vision processing approaches may be helpful in future OCT studies of ACHM (Hensel J., IOVS 2020;61:ARVO E-Abstract 3248).

### Limitations

Possible limitations of our study include the use of the qualitative EZ grading scale set by Sundaram et al., as it has a degree of subjectivity to it.^12^ This is important to point out, as all patients with ACHM have nystagmus, which can contribute to a reduced image quality and may complicate the assignment of OCT grades. The images included for assessment across follow-up used a mixed sample of vertical and horizontal line scans. This was done to bisect the fovea as accurately as possible and to use the highest quality scan possible. Although this should not affect the foveal ONL thickness value extracted for a given retina, any asymmetries in the EZ in the foveal region could result in different EZ grading (especially between grades II and IV). Furthermore, there was considerable variation in our follow-up periods, for example, our shortest follow up was 0.44 years (5 months) and our longest was 10 years (although this approach is typical for rare conditions like this). An important strength, however, is that we interpret the ONL thickness changes in context to our established repeatability of these measures in this population, allowing us to confidently ascribe changes as being “clinically significant” or within the measurement error.

It is important to note that our current analysis does not inform as to what cone structure will be in the future in our individuals – just that, in the period followed here, there was generally stable foveal cone structure. Ultimately, it is possible that cone structure could display a near-stable natural history for long periods in most patients with ACHM, followed by quick progression at some point later in life. Future monitoring of patients over time will help determine the frequency of any such progressive events.

## Conclusions

Continued studies and imaging will provide more data in understanding the progressive nature of ACHM. Research has illuminated on the initial concerns regarding efficacy of gene therapy due to cortical remapping and age.^56^ The hypothesis was that older individuals would have decreased cortical plasticity, but recent gene augmentation studies in individuals with ACHM have shown that this may not be the case with one study having a greater response in adults (>18 years old).^16^^,^^17^ A recent study looked at how loss of cone input affects cortical remapping using eccentricity and population field mapping (pRF).^57^ This study concluded that the absence of cone input may be preferred when it comes to gene therapy as a lack of cortical remapping supports better therapeutic outcomes (i.e. sensory conflicts) and a visual cortex response (i.e. pRF size reduction post gene therapy), as seen in McKyton et al.^17^ Furthermore, studies have found that they were able to recover dormant cone photoreceptor pathways in the visual cortex in individuals aged 10 to 15 years and that those with ACHM have greater cortical thickness at the fovea when compared to controls.^18^^,^^58^ Other findings from these gene augmentation studies include subjective improvements in photoaversion and color vision, and an ability to discern a color from grayscale background, providing promise for future treatment efforts.^17^^,^^18^ Although there may be examples of progressive alterations in retinal structure in some patients with ACHM, it is not definitive that such changes can be ascribed to the primary ACHM disease sequence. Whether or not this is the case could impact therapeutic response, so further elucidation of contributing factors to ACHM “progression” is imperative.

## Supplementary Material

Supplement 1

Supplement 2

Supplement 3

Supplement 4

## References

[1] Michaelides M, Hunt DM, Moore AT. The cone dysfunction syndromes. *Br J Ophthalmol**.* 2004; 88(2): 291–297. 14736794 10.1136/bjo.2003.027102 PMC1771989

[2] Kohl S, Zobor D, Chiang W, et al. Mutations in the unfolded protein response regulator ATF6 cause the cone dysfunction disorder achromatopsia. *Nat Genet**.* 2015; 47(7): 757–765. 26029869 10.1038/ng.3319 PMC4610820

[3] Johnson S, Michaelides M, Aligianis IA, et al. Achromatopsia caused by novel mutations in both CNGA3 and CNGB3. *J Med Genet**.* 2004; 41(2): e20. 14757870 10.1136/jmg.2003.011437 PMC1735666

[4] Kohl S, Varsanyi B, Antunes GA, et al. CNGB3 mutations account for 50% of all cases with autosomal recessive achromatopsia. *Eur J Hum Genet**.* 2005; 13(3): 302–308. 15657609 10.1038/sj.ejhg.5201269

[5] Aboshiha J, Dubis AM, Carroll J, Hardcastle AJ, Michaelides M. The cone dysfunction syndromes. *Br J Ophthalmol**.* 2016; 100(1): 115–121. 25770143 10.1136/bjophthalmol-2014-306505 PMC4717370

[6] Kohl S, Baumann B, Rosenberg T, et al. Mutations in the cone photoreceptor G-protein α-subunit gene GNAT2 in patients with achromatopsia. *Am J Hum Genet**.* 2002; 71(2): 422–425. 12077706 10.1086/341835 PMC379175

[7] Chang B, Grau T, Dangel S, et al. A homologous genetic basis of the murine cpfl1 mutant and human achromatopsia linked to mutations in the PDE6C gene. *Proc Natl Acad Sci USA**.* 2009; 106(46): 19581–19586. 19887631 10.1073/pnas.0907720106 PMC2780790

[8] Kohl S, Coppieters F, Meire F, et al. A nonsense mutation in PDE6H causes autosomal-recessive incomplete achromatopsia. *Am J Hum Genet**.* 2012; 91(3): 527–532. 22901948 10.1016/j.ajhg.2012.07.006 PMC3511981

[9] Ansar M, Santos-Cortez RLP, Saqib MAN, et al. Mutation of ATF6 causes autosomal recessive achromatopsia. *Hum Genet**.* 2015; 134(9): 941–950. 26063662 10.1007/s00439-015-1571-4 PMC4529463

[10] Genead MA, Fishman GA, Rha J, et al. Photoreceptor structure and function in patients with congenital achromatopsia. *Invest Ophthalmol Vis Sci**.* 2011; 52(10): 7298–7308. 21778272 10.1167/iovs.11-7762 PMC3183969

[11] Thomas MG, Kumar A, Kohl S, Proudlock FA, Gottlob I. High-resolution in vivo imaging in achromatopsia. *Ophthalmology**.* 2011; 118(5): 882–887. 21211844 10.1016/j.ophtha.2010.08.053

[12] Sundaram V, Wilde C, Aboshiha J, et al. Retinal structure and function in achromatopsia: implications for gene therapy. *Ophthalmology**.* 2014; 121(1): 234–245. 24148654 10.1016/j.ophtha.2013.08.017 PMC3895408

[13] Hood DC, Zhang X, Ramachandran R, et al. The inner segment/outer segment border seen on optical coherence tomography is less intense in patients with diminished cone function. *Invest Ophthalmol Vis Sci**.* 2011; 52(13): 9703–9709. 22110066 10.1167/iovs.11-8650 PMC3341126

[14] Michalakis S, Gerhardt M, Rudolph G, Priglinger S, Priglinger C. Achromatopsia: genetics and gene therapy. *Mol Diagn Ther**.* 2022; 26(1): 51–59. 34860352 10.1007/s40291-021-00565-z PMC8766373

[15] Fischer MD, Michalakis S, Wilhelm B, et al. Safety and vision outcomes of subretinal gene therapy targeting cone photoreceptors in achromatopsia: a nonrandomized controlled trial *JAMA Ophthalmol**.* 2020; 138(6): 643–651. 32352493 10.1001/jamaophthalmol.2020.1032 PMC7193523

[16] Michaelides M, Hirji N, Wong SC, et al. First-in-human gene therapy trial of AAV8-hCARp.hCNGB3 in adults and children with CNGB3-associated achromatopsia. *Am J Ophthalmol**.* 2023; 253: 243–251. 37172884 10.1016/j.ajo.2023.05.009

[17] McKyton A, Ohana DM, Nahmany E, Banin E, Levin N. Seeing color following gene augmentation therapy in achromatopsia. *Curr Biol**.* 2023; 33(16): 3489–3494.e3482. 37433300 10.1016/j.cub.2023.06.041

[18] Farahbakhsh M, Anderson EJ, Maimon-Mor RO, et al. A demonstration of cone function plasticity after gene therapy in achromatopsia. *Brain**.* 2022; 145(11): 3803–3815. 35998912 10.1093/brain/awac226 PMC9679164

[19] Thiadens AAHJ, Somervuo V, van den Born LI, et al. Progressive loss of cones in achromatopsia: an imaging study using spectral-domain optical coherence tomography. *Invest Ophthalmol Vis Sci**.* 2010; 51(11): 5952–5957. 20574029 10.1167/iovs.10-5680

[20] Thomas MG, McLean RJ, Kohl S, Sheth V, Gottlob I. Early signs of longitudinal progressive cone photoreceptor degeneration in achromatopsia. *Br J Ophthalmol**.* 2012; 96(9): 1232–1236. 22790432 10.1136/bjophthalmol-2012-301737

[21] Aboshiha J, Dubis AM, Cowing J, et al. A prospective longitudinal study of retinal structure and function in achromatopsia. *Invest Ophthalmol Vis Sci**.* 2014; 55(9): 5733–5743. 25103266 10.1167/iovs.14-14937 PMC4161486

[22] Zobor D, Werner A, Stanzial F, et al. The clinical phenotype of CNGA3-related achromatopsia: pretreatment characterization in preparation of a gene replacement therapy trial. *Invest Ophthalmol Vis Sci**.* 2017; 58(2): 821–832. 28159970 10.1167/iovs.16-20427

[23] Langlo CS, Erker LR, Parker M, et al. Repeatability and longitudinal assessment of foveal cone structure in CNGB3-associated achromatopsia. *Retina**.* 2017; 37(10): 1956–1966. 28145975 10.1097/IAE.0000000000001434 PMC5537050

[24] Hirji N, Georgiou M, Kalitzeos A, et al. Longitudinal assessment of retinal structure in achromatopsia patients with long-term follow-up. *Invest Ophthalmol Vis Sci**.* 2018; 59(15): 5735–5744. 30513534 10.1167/iovs.18-25452 PMC6280917

[25] Brunetti-Pierri R, Karali M, Melillo P, et al. Clinical and molecular characterization of achromatopsia patients: a longitudinal study. *Int J Mol Sci**.* 2021; 22(4): 1681. 33562422 10.3390/ijms22041681 PMC7914547

[26] Tekavčič Pompe M, Vrabič N, Volk M, et al. Disease progression in CNGA3 and CNGB3 retinopathy; characteristics of Slovenian cohort and proposed OCT staging based on pooled data from 126 patients from 7 studies. *Curr Issues Mol Biol**.* 2021; 43(2): 941–957. 34449556 10.3390/cimb43020067 PMC8929018

[27] Triantafylla M, Papageorgiou E, Thomas MG, et al. Longitudinal evaluation of changes in retinal architecture using optical coherence tomography in achromatopsia. *Invest Ophthalmol Vis Sci**.* 2022; 63(9): 6. 10.1167/iovs.63.9.6 PMC9363676 35930270

[28] Schneider CA, Rasband WS, Eliceiri KW. NIH Image to ImageJ: 25 years of image analysis. *Nat Methods**.* 2012; 9(7): 671–675. 22930834 10.1038/nmeth.2089 PMC5554542

[29] Thévenaz P, Ruttimann UE, Unser M. A pyramid approach to subpixel registration based on intensity. *IEEE Trans Image Process**.* 1998; 7(1): 27–41. 18267377 10.1109/83.650848

[30] Bland JM, Altman DG. Measuring agreement in method comparison studies. *Stat Methods Med Res**.* 1999; 8(2): 135–160. 10501650 10.1177/096228029900800204

[31] Langlo CS, Patterson EJ, Higgins BP, et al. Residual foveal cone structure in CNGB3-associated achromatopsia. *Invest Ophthalmol Vis Sci**.* 2016; 57(10): 3984–3995. 27479814 10.1167/iovs.16-19313 PMC4978151

[32] Mastey RR, Gaffney M, Litts KM, et al. Assessing the interocular symmetry of foveal outer nuclear layer thickness in achromatopsia. *Transl Vis Sci Tech**.* 2019; 8(5): 21. 10.1167/tvst.8.5.21 PMC6779097 31602346

[33] Scoles D, Sulai YN, Langlo CS, et al. In vivo imaging of human cone photoreceptor inner segments. *Invest Ophthalmol Vis Sci**.* 2014; 55(7): 4244–4251. 24906859 10.1167/iovs.14-14542 PMC4095721

[34] Litts KM, Woertz EN, Georgiou M, et al. Optical coherence tomography artifacts are associated with adaptive optics scanning light ophthalmoscopy success in achromatopsia. *Transl Vis Sci Tech**.* 2021; 10(1): 11. 10.1167/tvst.10.1.11 PMC7804582 33510950

[35] Litts KM, Georgiou M, Langlo CS, et al. Interocular symmetry of foveal cone topography in congenital achromatopsia. *Curr Eye Res**.* 2020; 45(10): 1257–1264. 32108519 10.1080/02713683.2020.1737138 PMC7487033

[36] Garrioch R, Langlo C, Dubis AM, et al. Repeatability of in vivo parafoveal cone density and spacing measurements. *Optom Vis Sci**.* 2012; 89(5): 632–643. 22504330 10.1097/OPX.0b013e3182540562 PMC3348369

[37] Jackson K, Vergilio GK, Cooper RF, Ying GS, Morgan JIW. A 2-year longitudinal study of normal cone photoreceptor density. *Invest Ophthalmol Vis Sci**.* 2019; 60(5): 1420–1430. 30943290 10.1167/iovs.18-25904 PMC6736277

[38] Dubis AM, Cooper RF, Aboshiha J, et al. Genotype-dependent variability in residual cone structure in achromatopsia: toward developing metrics for assessing cone health. *Invest Ophthalmol Vis Sci**.* 2014; 55(11): 7303–7311. 25277229 10.1167/iovs.14-14225 PMC4235328

[39] Mastey RR, Georgiou M, Langlo CS, et al. Characterization of retinal structure in ATF6-associated achromatopsia. *Invest Ophthalmol Vis Sci**.* 2019; 60(7): 2631–2640. 31237654 10.1167/iovs.19-27047 PMC6594318

[40] Georgiou M, Robson AG, Singh N, et al. Deep phenotyping of PDE6C-associated achromatopsia. *Invest Ophthalmol Vis Sci**.* 2019; 60(15): 5112–5123. 31826238 10.1167/iovs.19-27761 PMC6905659

[41] Yang P, Michaels KV, Courtney RJ, et al. Retinal morphology of patients with achromatopsia during early childhood: implications for gene therapy. *JAMA Ophthalmol**.* 2014; 132(7): 823–831. 24676353 10.1001/jamaophthalmol.2014.685 PMC8174570

[42] Hendrickson A, Possin D, Vajzovic L, Toth CA. Histologic development of the human fovea from midgestation to maturity. *Am J Ophthalmol**.* 2012; 154(5): 767–778. 22935600 10.1016/j.ajo.2012.05.007 PMC3509500

[43] Dubis AM, Costakos DM, Subramaniam CD, et al. Evaluation of normal human foveal development using optical coherence tomography and histologic examination. *Arch Ophthalmol**.* 2012; 130(10): 1291–1300. 23044942 10.1001/archophthalmol.2012.2270 PMC3724218

[44] Lee H, Purohit R, Sheth V, et al. Retinal development in infants and young children with achromatopsia. *Ophthalmology**.* 2015; 122(10): 2145–2147. 25972256 10.1016/j.ophtha.2015.03.033 PMC4582068

[45] Vajzovic L, Rothman AL, Tran-Viet D, et al. Delay in retinal photoreceptor development in very preterm compared to term infants. *Invest Ophthalmol Vis Sci**.* 2015; 56(2): 908–913. 25587063 10.1167/iovs.14-16021 PMC4321398

[46] O'Sullivan M, Ying G-S, Mangalesh S, et al. Foveal differentiation and inner retinal displacement are arrested in extremely premature infants. *Invest Ophthalmol Vis Sci**.* 2021; 62(2): 25. 10.1167/iovs.62.2.25 PMC7900865 33599735

[47] He Y, Chen X, Tsui I, Vajzovic L, Sadda SR. Insights into the developing fovea revealed by imaging. *Prog Retin Eye Res**.* 2022; 90(101067. 35595637 10.1016/j.preteyeres.2022.101067 PMC12183701

[48] Grossniklaus HE, Nickerson JM, Edelhauser HF, Bergman LA, Berglin L. Anatomic alterations in aging and age-related diseases of the eye. *Invest Ophthalmol Vis Sci**.* 2013; 54(14): ORSF23–ORSF27. 24335063 10.1167/iovs.13-12711 PMC3864374

[49] Trinh M, Khou V, Zangerl B, Kalloniatis M, Nivison-Smith L. Modelling normal age-related changes in individual retinal layers using location-specific OCT analysis. *Sci Rep**.* 2021; 11(1): 558. 33436715 10.1038/s41598-020-79424-6 PMC7804110

[50] Jacobson SG, Aleman TS, Cideciyan AV, et al. Human cone photoreceptor dependence on RPE65 isomerase. *Proc Natl Acad Sci USA**.* 2007; 104(38): 15123–15128. 17848510 10.1073/pnas.0706367104 PMC1986623

[51] Demirkaya N, van Dijk HW, van Schuppen SM, et al. Effect of age on individual retinal layer thickness in normal eyes as measured with spectral-domain optical coherence tomography. *Invest Ophthalmol Vis Sci**.* 2013; 54(7): 4934–4940. 23761080 10.1167/iovs.13-11913 PMC5963176

[52] Curcio CA, Millican CL, Allen KA, Kalina RE. Aging of the human photoreceptor mosaic: evidence for selective vulnerability of rods in central retina. *Invest Ophthalmol Vis Sci**.* 1993; 34(12): 3278–3296. 8225863

[53] Shelley EJ, Madigan MC, Natoli R, Penfold PL, Provis JM. Cone degeneration in aging and age-related macular degeneration. *Arch Ophthalmol**.* 2009; 127(4): 483–492. 19365029 10.1001/archophthalmol.2008.622

[54] Panda-Jonas S, Jonas JB, Jakobczyk-Zmija M. Retinal photoreceptor density decreases with age. *Ophthalmology**.* 1995; 102(12): 1853–1859. 9098287 10.1016/s0161-6420(95)30784-1

[55] Georgiou M, Singh N, Kane T, et al. Long-term investigation of retinal function in patients with achromatopsia. *Invest Ophthalmol Vis Sci**.* 2020; 61(11): 38. 10.1167/iovs.61.11.38 PMC7509756 32960951

[56] Baseler HA, Brewer AA, Sharpe LT, et al. Reorganization of human cortical maps caused by inherited photoreceptor anomalies. *Nat Neurosci**.* 2002; 5(4): 364–370. 11914722 10.1038/nn817

[57] Molz B, Herbik A, Baseler HA, et al. Achromatopsia-visual cortex stability and plasticity in the absence of functional cones. *Invest Ophthalmol Vis Sci**.* 2023; 64(13): 23. 10.1167/iovs.64.13.23 PMC10584018 37847226

[58] Molz B, Herbik A, Baseler HA, et al. Structural changes to primary visual cortex in the congenital absence of cone input in achromatopsia. *Neuroimage Clin**.* 2022; 33: 102925. 34959047 10.1016/j.nicl.2021.102925 PMC8718719

[59] Wells-Gray EM, Choi SS, Bries A, Doble N. Variation in rod and cone density from the fovea to the mid-periphery in healthy human retinas using adaptive optics scanning laser ophthalmoscopy. *Eye**.* 2016; 30(8): 1135–1143. 27229708 10.1038/eye.2016.107 PMC4985666

