# Supplementary material for: Longitudinal Assessment of OCT-Based Measures of Foveal Cone Structure in Achromatopsia
Source: Invest Ophthalmol Vis Sci. 2024 Apr 8;65(4):16. doi: 10.1167/iovs.65.4.16 (PMC11005076; doi:10.1167/iovs.65.4.16)
Supplement: Supplement 1 [file iovs-65-4-16_s001.pdf]

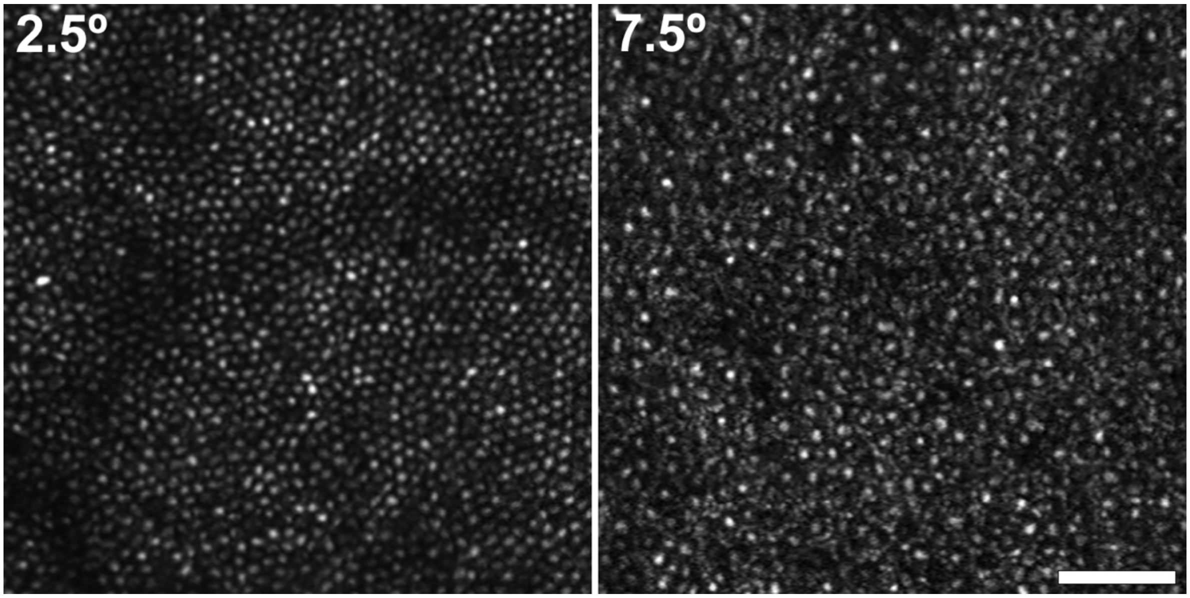

Supplementary Figure S1. Confocal AOSLO images of the photoreceptor mosaic from a 39-year-old male with normal vision. Scale bar = 20 μm.
