# Supplementary material for: Longitudinal Assessment of OCT-Based Measures of Foveal Cone Structure in Achromatopsia
Source: Invest Ophthalmol Vis Sci. 2024 Apr 8;65(4):16. doi: 10.1167/iovs.65.4.16 (PMC11005076; doi:10.1167/iovs.65.4.16)
Supplement: Supplement 3 [file iovs-65-4-16_s003.pdf]

Supplementary Table S2. Baseline Characteristics & Genotype Information				
ID	Gene	Pathogenic Variant	Sex	Age at Baseline (years)
JC_0046	*CNGA3*	c.1709G>T:p.Ser570Ile*	M	51.12
JC_0047	*CNGB3*	c.1148delC:p.Thr383Ilefs*	M	11.70
JC_0056	*CNGB3*	c.1148delC:p.Thr383Ilefs*	M	50.16
JC_0185	*CNGA3*	c.985G>T:p.Glyc329Cys; c.1306C>T:p.Arg436Trp	F	16.10
JC_0605	*CNGB3*	c.1148del:p.Thr383Ilefs*13; c.1578+1G>A	F	30.81
JC_0686	*CNGB3*	Thr383 del1aC*	F	48.26
JC_0793	*CNGA3*	c.1580T>G;p.L527R; c.829C>T;p.R277C	F	15.26
JC_0794	*CNGA3*	c.829C>T;p.R277C; c.1580T>G;p.L527R	M	12.14
JC_10008	*CNGA3*	c.829C>CT; c.1641C>AC	F	12.54
JC_10024	*CNGB3*	c.1148delC:p.Thr383Ilefs*	M	26.62
JC_10025	*CNGB3*	c.1148delC:p.Thr383Ilefs*	M	18.52
JC_10028	*CNGB3*	c.1148delC:p.Thr383Ilefs*	F	13.24
JC_10029	*CNGB3*	c.1148delC:p.Thr383Ilefs*	F	11.37
JC_10069	*CNGA3*	p.Arg283Trp:c.847C>T; p.Tyr181Cys:c.542A>G	M	18.09
JC_10089	*CNGB3*	c.1148delC:p.Thr383Ilefs*	F	40.04
JC_10142	*CNGB3*	c.1148delC:p.Thr383Ilefs; c.983T>A:p.Met328Lys	F	44.68
JC_10151	*CNGB3*	c.1148delC:p.Thr383Ilefs; c.503C>T:p.Thr168Met; c.1602T>G:p.Tyr534Ter	F	10.47
JC_10167	*CNGB3*	c.1148delC:p.Thr383Ilefs; c.1255G>T:p.Glu419Ter	F	16.72
JC_10168	*CNGB3*	c.1148delC:p.Thr383Ilefs*	F	9.23
JC_10191	*CNGB3*	c.1148delC:p.Thr383Ilefs*	M	35.76
JC_10195	*CNGB3*	c.1148delC:p.Thr383Ilefs*	M	8.23
JC_10196	*CNGB3*	c.1148delC:p.Thr383Ilefs*	F	33.10
JC_10197	*CNGB3*	c.1148delC:p.Thr383Ilefs; c.1320þ4A>G**	F	8.82
JC_10198	*CNGB3*	c.1148delC:p.Thr383Ilefs*	F	43.62
JC_10213	*CNGB3*	c.1148delC:p.Thr383Ilefs; c.886_896del11insT:p.Arg296Tyrfs	M	11.03
JC_10215	*CNGB3*	c.1148delC:p.Thr383Ilefs*	M	13.43
JC_10216	*CNGB3*	c.1148delC:p.Thr383Ilefs; c.819_826del8:p.Arg274Valfs	M	17.17
JC_10217	*CNGB3*	c.1148delC; c.819_826delCAC-ACTCC	F	7.94

JC_10224	*CNGB3*	c.1148delC:p.Thr383Ilefs; c.983T>A:p.Met328Lys	M	37.53
JC_10226	*CNGB3*	c.1148delC:p.Thr383Ilefs*	F	38.05
JC_10232	*CNGB3*	c.1148delC:p.Thr383Ilefs*	M	18.23
JC_10247	*CNGB3*	c.1148delC:p.Thr383Ilefs*	M	24.13
JC_10248	*CNGB3*	c.1148delC:p.Thr383Ilefs*	F	16.08
JC_10249	*CNGB3*	c.1148delC:p.Thr383Ilefs*	M	15.46
JC_10250	*CNGB3*	c.1148delC:p.Thr383Ilefs*	M	17.18
JC_10256	*CNGB3*	c.1148delC:p.Thr383Ilefs*	F	22.13
JC_10257	*CNGB3*	c.1148delC:p.Thr383Ilefs*	M	14.61
JC_10258	*CNGB3*	c.1148delC:p.Thr383Ilefs*	F	42.70
JC_10260	*CNGB3*	c.1148delC:p.Thr383Ilefs*	M	31.62
JC_10300	*CNGB3*	c.819_826del8; c.1148delC	M	8.15
JC_10301	*CNGB3*	c.1148delC:p.Thr383Ilefs*	F	6.80
JC_10310	*CNGB3*	c.1148delC:p.Thr383Ilefs; c.819_826del8:p.Arg274Valfs	M	33.3
JC_10320	*CNGB3*	c.1148delC:p.Thr383Ilefs*	M	12.97
JC_10334	*CNGB3*	c.1148delC:p.Thr383Ilefs*	M	22.00
JC_10409	*CNGB3*	c.1148delC:p.Thr383Ilefs*	F	32.49
JC_10416	*CNGB3*	c.1148delC:p.Thr383Ilefs*	F	30.39
JC_10417	*CNGB3*	c.1148delC:p.Thr383Ilefs; c.1751T>C:p.Leu584Pro	F	23.16
JC_10424	*CNGB3*	c.819_826del8:p.Arg274Valfs; c.1781þ1G>C: splice defect	M	27.14
JC_10494	*CNGB3*	c.1148delC:p.Thr383Ilefs; c.1180T>A:p.Tyr394Asn	M	27.55
JC_10617	*CNGA3*	c.1641C>A:p.Phe547Leu*	M	14.14
JC_10853	*CNGB3*	c.1148delC:p.Thr383Ilefs*	F	28.8
JC_10854	*CNGB3*	c.1148delC:p.Thr383Ilefs*	M	32.22
JC_10953	*CNGB3*	c.1148delC:p.Thr383Ilefs*	M	16.48
JC_10966	*CNGB3*	c.1148delC:p.Thr383Ilefs*	M	8.55
JC_10968	*CNGB3*	c.1148delC:p.Thr383Ilefs*	M	43.89
JC_10999	*CNGB3*	c.1148delC:p.Thr383Ilefs*	F	9.94
JC_11036	*CNGB3*	c.1148delC:p.Thr383Ilefs*	F	7.69
JC_11061	*CNGB3*	c.1148delC:p.Thr383Ilefs*	F	7.41
JC_11091	*CNGB3*	c.1148delC:p.Thr383Ilefs*	F	32.03
JC_1184	*CNGB3*	c.1148delC:p.Thr383Ilefs; c.1006G>T:p.Glu336Ter	M	22.05
JC_1208	*CNGB3*	c.1148delC:p.Thr383Ilefs*	M	16.01
KS_10088	*CNGA3*	c.450-1G>A; c.1557G>A:p.Met519Ile	F	64.11
TM_11446	*ATF6*	c.970C>T:p.Arg324Cys; c.1664G>C:p.Arg555Thr	M	9.62

*Participant is homozygous for pathogenic variant
