# Supplementary material for: Longitudinal Assessment of OCT-Based Measures of Foveal Cone Structure in Achromatopsia
Source: Invest Ophthalmol Vis Sci. 2024 Apr 8;65(4):16. doi: 10.1167/iovs.65.4.16 (PMC11005076; doi:10.1167/iovs.65.4.16)
Supplement: Supplement 4 [file iovs-65-4-16_s004.pdf]

Supplementary Methods

Adaptive Optics Scanning Light Ophthalmoscopy (AOSLO)

Confocal and split-detector AOSLO images were simultaneously obtained using a previously described custom-built AOSLO system.[1] In brief, image sequences were acquired using both a 1° × 1° and 1.75° × 1.75° field of view imaging raster (790nm). The participant was instructed to follow a fixation target to bring different retinal locations into view and allow collection of overlapping retinal locations across the parafovea. Sinusoidal distortions in the raw images were corrected by measuring the distortion with a Ronchi ruling of known spacing and resampling the images. The individual image sequences were then processed as follows. First, a reference frame with minimal distortion was selected from an image sequence,[2] and the remaining frames were broken up into strips and aligned to the reference frame based on normalized cross correlation, as previously described.[3] The registered image sequences were then averaged to improve the signal-to-noise ratio, and this process resulted in a single confocal TIFF image and a single split-detection TIFF image for each image sequence. The TIFF images were then automatically montaged using a previously described algorithm.[4] The output montage was imported into Photoshop (Adobe Photoshop; Adobe Systems, Inc., San Jose, CA, USA) and alignments were manually inspected and adjusted as needed. The image scale was calculated by first determining the degrees per pixel in an image of a ruling with known spacing using the small angle approximation. This value was then linearly scaled using the participant's axial length and a reference axial length of 24 mm and multiplied by the retinal magnification factor of 291 μm/degree to obtain a final micrometer per pixel scale. Analysis of cone spacing was performed using previously described methods.[5]

References:

1. Langlo CS, Patterson EJ, Higgins BP, et al. Residual foveal cone structure in *CNGB3*-associated achromatopsia. *Invest Ophthalmol Vis Sci* 2016;57:3984-3995.
2. Salmon AE, Cooper RF, Langlo CS, Baghaie A, Dubra A, Carroll J. An automated reference frame selection (ARFS) algorithm for cone imaging with adaptive optics scanning light ophthalmoscopy. *Transl Vis Sci Technol* 2017;6:9.
3. Dubra A, Harvey Z. Registration of 2D images from fast scanning ophthalmic instruments. In: Fischer B, Dawant B, Lorenz C (eds), *Biomedical Image Registration.* Berlin: Springer-Verlag; 2010:60-71.
4. Chen M, Cooper RF, Han GK, Gee J, Brainard DH, Morgan JI. Multi-modal automatic montaging of adaptive optics retinal images. *Biomed Opt Express* 2016;7:4899-4918.
5. Cooper RF, Wilk MA, Tarima S, Carroll J. Evaluating descriptive metrics of the human cone mosaic. *Invest Ophthalmol Vis Sci* 2016;57:2992-3001.
